# Malignant Lymphomas

**DOI:** 10.1038/sj.bjc.6600426

**Published:** 2002-08-01

**Authors:** Peter Amlot

**Affiliations:** Royal Free and University College Medical School, London, UK

## Abstract

*British Journal of Cancer* (2002) **87**, 372. doi:10.1038/sj.bjc.6600426 www.bjcancer.com

© 2002 Cancer Research UK

## 

In this age of Internet access to vast online medical reference databases, one has to ask what encourages the ownership of medical books. Three outstanding reasons are the wish to have a comprehensive reference work, an introduction to a new subject or the analysis or metanalysis of an overcrowded literature. ‘Malignant Lymphomas’ in the American Cancer Society series belongs to the last of these three categories. It is not a reference work, which is surprising for a series entitled the ‘Atlas of Clinical Oncology’. For a reference work there are just too many missing topics that one would expect to have been covered. Some lymphomas, such as chronic lymphocytic leukaemia, hairy cell leukaemia, adult T cell leukaemia/lymphoma or post-transplant lymphoproliferative disease may have been left to be dealt with by other books in the series. However, the absence of lymphoplasmacytoid lymphoma, myeloma, splenic marginal zone and anaplastic large cell lymphoma can hardly be regarded as oversights and the lack of any coverage of the T cell lymphomas except for mycosis fungoides/Sezary's syndrome is a glaring omission. On the other hand, it is too detailed to be an introduction to malignant lymphomas for the novice.

What ‘Malignant Lymphomas’ covers, with a few exceptions, are the commonest types of B cell lymphoma and Hodgkin's disease. The first chapter deals with histopathology of lymphomas and this chapter underlines how many lymphoma categories are missing from this book. It also illustrates one of the problems within the structure of the book, a perennial problem for non-Hodgkin's lymphoma, which is lymphoma classification. The recent WHO classification, based on the REAL classification, has been chosen as the basis upon which ‘Malignant Lymphomas’ should be analysed. Clinicians have become so nervous about lymphoma classification, that almost every chapter thereafter gives a potted version of lymphoma histopathology and being unprepared to analyse their clinical data in terms of the new classification usually revert thereafter to the use of broad categories such as ‘Indolent’ and ‘Aggressive’ lymphomas, neither of which should have any place in the WHO classification. The photomicrographs used to illustrate pathology in the first chapter are generally too underexposed but usually interpretable whereas in other chapters they can be uninterpretable because of the underexposure. The following chapters on Epidemiology and Molecular Biology adequately cover the scientific basis of lymphomas while chapters on Staging and Prognostic factors cover the clinical science. Thereafter the analysis of lymphomas is largely broken down into three main clinical categories – Indolent, Diffuse Large Cell and High-Grade lymphomas with two chapters devoted to the special circumstances of lymphomas in AIDS and the elderly. There are odd chapters on MALT, mantle cell and primary mediastinal lymphomas but these seem to be special side-shows rather than part of a systematic coverage. It is odd that neither the Epidemiology nor MALT chapters examine the aetiological and biological significance of Helicobacter pylori. The management of the three main clinical sections is dealt within the chapters attributed to them and then special aspects are revisited by chapters on High Dose Therapy, Stem Cell Transplantation, Salvage Chemotherapy and Monoclonal antibody therapy. These sections overlap each other and continue to undermine any attempt to make the subject consistent with the WHO classification. Although there is a wealth of information and reference in these clinical chapters, it leaves one with the strong impression that they are based around the expertise of individual authors rather than a systematic, science driven plan. This lack of co-ordination and integration has to be due to the editorial policy behind the book and ought to be reconsidered in future editions.

In essence this book has been mainly constructed to put into biological context a review of clinical trials and lymphoma management using different cytotoxic chemotherapy regimes with or without autologous or allogeneic stem cell rescue. There is a dearth of any information peripheral to the therapeutic management of lymphomas. For example, the immunological effects of lymphoma, such as autoimmune haemolytic anaemia, immunodeficiency or opportunistic infection are not even mentioned. Even the management or strategy for dealing with neutropoenic sepsis is not addressed outside of the inclusion of G-CSF into drug regimens to prevent neutropoenia. In this way the book is very narrowly focussed and will consequently provide a useful summary of the current status of therapy for common lymphomas for medical, clinical and haemato-oncologists.

In conclusion, this book is suitable for those who wish to get a concise scientific background to lymphomas and obtain a fairly up to date review of the medical oncological management of the common clinical forms of lymphoma and Hodgkin's disease.

